# Household point of care CD4 testing and isoniazid preventive therapy initiation in a household TB contact tracing programme in two districts of South Africa

**DOI:** 10.1371/journal.pone.0192089

**Published:** 2018-03-02

**Authors:** Liesl Page-Shipp, James J. Lewis, Kavindhran Velen, Sedikanelo Senoge, Elizabeth Zishiri, Flora Popane, Violet N. Chihota, Dave Clark, Gavin J. Churchyard, Salome Charalambous

**Affiliations:** 1 The Aurum Institute, Johannesburg, South Africa; 2 London School of Hygiene and Tropical Medicine, London, United Kingdom; 3 The School of Public Health, University of Witwatersrand, Johannesburg, South Africa; 4 Advancing Care and Treatment for TB and HIV, Medical Research Council Collaborating Centre of Excellence, Johannesburg, South Africa; University of Cape Town, SOUTH AFRICA

## Abstract

**Background:**

In South Africa, TB household contact tracing provides an opportunity for increased TB and HIV case finding. We aimed to determine the effect of two new potential interventions for TB contact tracing programmes: Point of Care CD4 (PoC CD4) on HIV linkage to care and household Isoniazid Preventive Therapy (IPT) provision on uptake and retention of IPT.

**Methods:**

A pragmatic, three-arm, cluster-randomized trial was undertaken. TB Household contacts were randomised to 3 arms: 1) Standard of Care TB and HIV testing (SOC); 2) SOC with POC CD4 for those testing HIV positive; 3) SOC with POC CD4 and IPT for eligible household members. Linkage to care within 90 days was assessed either through patient visits (at 10 weeks and 6 months) or via telephonic contact.

**Results:**

2,243 index TB patients and 3,012 contacts (64,3% female, median age 30 years) were enrolled. On self-report, 26(1.2%) were currently receiving TB treatment and 1816 (60.3%) reported a prior HIV test. HIV testing uptake was 34.7% in the SoC arm, 40.2% in the PoC CD4 arm (RR1.16, CI 0.99–1.36, p-value = 0.060) and 39.9% in the PoC CD4 + HH-IPT arm (RR = 1.15, CI 0.99–1.35, p-value = 0.075). Linkage to care within 3 months was 30.8% in the SoC arm and 42.1% in the POC CD4 arms (RR 1.37; CI: 0.68–2.76, p-value = 0.382). 20/21 contacts (95.2%) initiated IPT in the PoC CD4 + HH-IPT arm, compared to 3/20 (15.0%) in the PoC CD4 arm (p = 0.004; p-value from Fisher’s exact test < 0.001). Among 3,008 contacts screened for tuberculosis, 15 (3.4%) had bacteriologically confirmed TB with an overall yield of TB of 0.5% (95% CI: 0.3%, 0.8%).

**Conclusions:**

Household PoC CD4 testing and IPT initiation is feasible. There was only weak evidence that PoCCD4 led to a small increase in HCT uptake and no evidence for an increase in linkage-to-care. IPT initiation and completion was increased by the household intervention. Although feasible, these interventions had low impact due to the low uptake of HIV testing in households.

## Introduction

UNAIDS has set an ambitious target to help end the AIDS epidemic: by 2020, 90% of all people living with HIV will know their HIV status; 90% of those diagnosed with HIV infection will receive sustained antiretroviral therapy and 90% of these will have viral suppression[1]. The inclusion of HIV testing for vulnerable populations is essential to meeting the first 90. Reaching the second pillar of ART initiation requires seemless linkage to care. Attrition along the HIV care pathway is a significant challenge with a recent review reporting that up to 28% of patients are lost between receiving a positive HIV result and CD4 count testing [2]. Furthermore, another systematic review indicated that approximately 43% of eligible patients are lost to ART initiation after CD4 testing in Sub-Saharan Africa[3].

Household contact tracing (HHCT) for TB is effective for TB Case finding [4–6] and forms part of the South African National Department of Health (NDOH) Primary Health Care Reengineering strategy [7] and the World Health Organisation (WHO) recommendations for systematic screening [8]. It also provides an opportunity to assist with meeting the ambitious UNAIDS HIV target, due to the high levels of HIV co-infection in TB patients[9]; and high HIV prevalence in TB affected households[5,6,10]. Ward-Based outreach teams (WBOT) comprised of Community Health Workers (CHWs) are part of this strategy. This diverse cadre of staff are expected to fulfil multiple tasks and understanding how HH HIV testing can efficiently be deployed may help inform the WBOT programme.

Point of care CD4 (PoC CD4) testing resulted in a three-fold increase in ART initiation compared to standard of care in an urban primary health care facility in South Africa[11]. In community settings, PoC CD4 has been shown to increase the likelihood that patients would visit a referral centre after receiving community mobile HIV testing [12] and Wynberg et al reviewed three mobile clinic PoC CD4 studies and suggested a role for PoC testing in community and household testing programmes [13]. It was therefore postulated that offering PoC CD4 in the household would increase linkage to care.

Isoniazid Preventive Therapy (IPT) is recognised to be effective in preventing TB, particularly in Tuberculin Skin Test positive individuals [14]. The need to scale-up IPT is well recognised [15] and different strategies need to be explored. Household IPT initiation may increase uptake of IPT as individuals identified with HIV in the household setting are more likely to have less advanced disease[16]; be in the previously defined ‘‘pre-ART” phase and would not otherwise seek care. In addition, household IPT implementation may remove the need, inconvenience and expense of clinic visits and potential exposure to TB through these visits. In addition, a prerequisite to starting IPT is TB screening, HIV testing and CD4 staging, which are all part of the HHCT strategy, these visits could thus be used as an entry point to household IPT initiation for eligible contacts. Household IPT initiation has not previously been described.

The **aim** of this study was to determine the effect of PoC CD4 on HIV entry into care and to determine the effect of household IPT provision on uptake and retention of IPT in eligible patients.

## Methods

### Study design

A pragmatic, three-arm, cluster-randomized trial with the randomization occurring at household level.

### Study setting

The study was undertaken among household contacts of index TB patients in two districts in South Africa: Sekhukhune District in Limpopo Province is largely rural and Ekurhuleni District in Gauteng Province is urbanized. Index TB patients > 18 years old were recruited from primary health care clinics where they were receiving their routine TB care. Patients who had a positive smear, Xpert MTB/RIF (Xpert) or culture result were invited to participateand signed an informed consent for their household members to be contacted. A household contact was defined as any individual, regardless of age, who had been in contact with the index patient and who generally slept and shared meals in the same household.

### Randomisation

A list of study numbers for index TB patients and their household members was generated at the start of the study, with each study number including the arm to which the patient would be assigned, generated randomly using the random number function “rand()” in Excel. Consecutively recruited patients were allocated the first unallocated study number on the list, thus randomising them to study arm.

### Interventions

Households were randomised into three arms. The first arm (“SoC arm”) offered Standard of Care HIV counselling and testing to all contacts ≥ 14 years and to contacts <14 if the mother self-reported to be HIV positive or tested positive during the visit. Any HIV positive patient currently not in care, whether newly diagnosed or self-reported HIV-positive, was given an appropriate referral letter. As part of TB contact tracing, participants > 5 years received a verbal four symptom TB symptom screen and those with any symptom suggestive of TB were assisted to provide sputum for laboratory testing through the routine national laboratory service. Participants with TB positive results were contacted and advised to attend their local clinic for TB treatment.

Although children < 5 years were not offered the randomised intervention, consent was requested from the care-giver for retrospective record review and they were offered standard of care according to National guidelines, which is referral to the nearest clinic, either for initiation of TB treatment or IPT.

The second arm (“PoC CD4 arm”) included all interventions above with the addition of a rapid PoC CD4 test for patients aged at least 14 years who were diagnosed as HIV-positive at the baseline household visit. Those with CD4< 350 were advised that they were eligible for ART and were given an immediate referral for ART.

The third arm (“PoC CD4 + HH-IPT arm”) included all interventions described above. In addition contacts with a CD4 of <350 were supplied with cotrimoxazole (CMX) on a monthly basis for three months and immediately referred. Participants with CD4 ≥350 were offered household IPT (HH-IPT) for six months if they had no TB symptoms or other contraindications. Those who accepted HH-IPT were given a one month supply of isoniazid (INH) and then followed-up monthly in the household for six months to evaluate development of TB symptoms, INH side effects and to issue another one month supply of INH.

If participants did not consent to participate in the randomisation intervention, they were nevertheless offered standard of care. For those who agreed, written informed consent was obtained from adult index patients and household contacts. For participants <18 years old, an assent form was completed with a consent form signed by a parent or guardian.

### Follow up

All enrolled contacts were revisited at ten weeks and six months to determine whether they had linked to HIV care (SoC and PoC CD4 arms), as well as whether they had initiated and continued taking IPT (PoC CD4 and PoC CD4 + HH-IPT arms). They were also re-screened for TB and offered HIV testing if appropriate. An attempt was made in March 2015 to telephone all contacts that were newly diagnosed as HIV-positive at the baseline visit, for whom we did not have a follow-up visit, to determine whether they had linked to care and/or initiated IPT.

### Endpoints

Linkage into care was defined as participant self-report of accessing a government or private clinic for repeat HIV or CD4 testing and/or for ART care, regardless of the outcome of the visit. Linkage must have occurred within 90 days of the baseline visit and was assessed at each of the ten week visit, the six month visit or via telephonic contact at the end of the study.

Co-primary endpoints were (1) the proportion of those with newly diagnosed HIV who linked to care within three months, comparing the SoC and PoC CD4 arms; and (2) the proportion of those with newly diagnosed HIV and a PoC CD4>350 who initiated IPT during follow-up, comparing the PoC CD4 and PoC CD4 + HH-IPT arms. The first co-primary endpoint was only compared between the SoC and PoC CD4 arms as the effect of the availability of household IPT for those with a CD4>350 in the PoC CD4 + HH-IPT arm could not be determined. Secondary endpoints were (1) uptake of HIV testing among those who did not who did not self-report being HIV-positive, comparing the three arms; (2) yield of newly diagnosed HIV among all household contacts > 14 years, comparing the three arms; and (3) the proportion of those with newly diagnosed HIV and a PoC CD4>350 who received at least four months of IPT, comparing the PoC CD4 and PoC CD4 + HH-IPT arms. Completion of four rather than six months of IPT, was used as this endpoint to allow for some delay in initiating IPT at clinics as study follow up time only allowed us to follow all patients to four months following enrollment.

### Statistical analysis

Comparisons between arms were presented as risk or prevalence ratios (with 95% confidence intervals and p-values) calculated from a generalised linear model with a binomial variance distribution, a log link function and robust standard errors calculated at the household level. All analyses are restricted to contacts aged at least 14 years old. Adjusted analyses were conducted that adjusted for age and gender *a priori*; adjustment was not made for other factors as they showed reasonable baseline balance and statistical power was low.

### Ethical approvals and trial registration

Ethical approval was obtained from the Research Ethics Committees of the University of the Witwatersrand, South Africa and the London School of Hygiene and Tropical Medicine. The study was registered with Current Controlled Trials (ISRCTN88864357).

## Results

### Demographics

**Fig 1:**
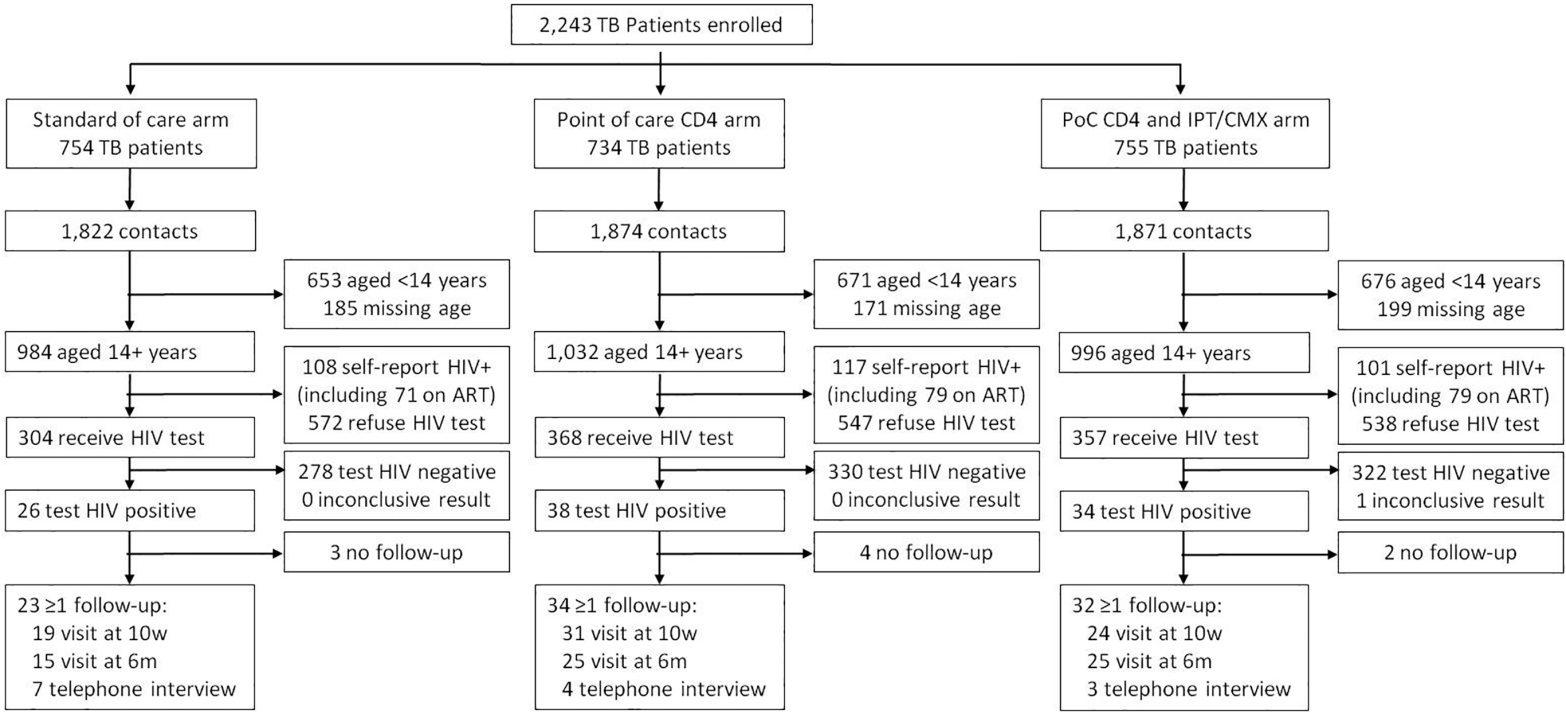
CONSORT diagram.

From November 2012 to August 2014, 2,243 index TB patients were enrolled into the study and randomised to one of three arms: 754 to the SoC arm, 734 to the PoCCD4 arm and 755 in the PoC CD4 +HH-IPT arm (Fig 1). There were 5,567 household contacts enrolled from these index TB patients: 1,822 (984 aged 14+ years old) in the SoC arm, 1,874 (1,032 aged 14+ years old) in the PoCCD4 arm and 1,871(996 aged 14+ years old) in the PoCCD4 +HH-IPT arm. The majority of contacts were female (63.2%; Table 1), median age was 30 years (inter-quartile range: 21, 50), 8.2% self-reported prior TB treatment, 1.2% self-reported current TB treatment and 60.3% self-reported a prior HIV test. There was reasonable baseline balance in these characteristics across the three study arms (Table 1). None of the contacts in the SoC arm received a PoC CD4 result, while 32 of 38 in the PoC CD4 arm and 28 of 34 in the PoC CD4 + HH-IPT arm received a PoC CD4 result (84% and 82% respectively; Table 2).

**Table 1:** Baseline characteristics for TB contacts aged 14+ years old and for those contacts in the primary analysis (aged 14+ years and newly diagnosed HIV-positive), by study arm.

	SoC arm (N = 984)	PoC CD4 arm (N = 1032)	PoC CD4 + HH-IPT arm (N = 996)
	n/N*(%)	n/N* (%)	n/N* (%)
**Contacts aged 14+ years old**			
Gauteng province	752/984 (76.4%)	809/1,032 (78.4%)	777/996 (78.0%)
Male	351/982 (35.7%)	384/1,031 (37.3%)	371/994 (37.3%)
Age (median; IQR)	30 (21–51)	31 (21–48)	30 (21–50)
Previous history of TB	66/976 (6.8%)	109/1,024 (10.6%)	70/989 (7.1%)
Currently taking TB treatment	8/970 (0.8%)	18/1,023 (1.8%)	10/987 (1.0%)
Prior HIV test	584/984 (59.4%)	637/1,032 (61.7%)	595/996 (59.7%)
Self-report HIV positive	108/984 (11.0%)	117/1,032 (11.3%)	101/996 (10.1%)
Self-report on ART	71/984 (7.2%)	79/1,032 (7.7%)	79/996 (7.9%)
**Contacts aged 14+ years old and newly diagnosed HIV positive(N = 98)**			
Gauteng province	23/26 (88.5%)	35/38 (92.1%)	31/34 (91.2%)
Male	5/26 (19.2%)	11/38 (29.0%)	13/34 (38.2%)
Age (median; IQR)	34.5 (29–48)	35.5 (28–44)	29 (23–44)
Previous history of TB	1/26 (3.9%)	3/38 (7.9%)	2/34 (5.9%)
Currently taking TB treatment	0/25 (0.0%)	0/38 (0.0%)	1/33 (3.0%)
Prior HIV test	16/26 (61.5%)	21/37 (56.8%)	22/34 (64.7%)

*Denominators may differ due to missing data

SoC = standard of care; PoC = point-of-care; HH-IPT = household isoniazid preventive therapy

**Table 2:** Point-of-care CD4 results for those newly diagnosed HIV-positive, by study arm.

CD4 result	SoC arm (arm 1) N = 26	PoC CD4 arm (arm 2) N = 38	PoC CD4+ HH-IPT arm (arm 3) N = 34
<350	0 (0.0%)	12 (31.6%)	7 (20.6%)
350–499	0 (0.0%)	11 (29.0%)	5 (14.7%)
500+	0 (0.0%)	9 (23.7%)	16 (47.1%)
Not done	26 (100.0%)	6 (15.8%)	6 (17.7%)

### HIV testing uptake

Across the three arms, 326 of 3,012 contacts aged 14+ years (10.8%) self-reported being HIV-positive, of whom 70.2% self-reported being on ART (similar across arms; Table 1 and Fig 1). Among the 2,686 who did not self-report being HIV-positive, 1,029 (38.3%) agreed to, and received, an HIV test (Fig 1). In the SoC arm, HIV test uptake was 34.7% (Table 3 and Fig 1). This was higher in both the PoC CD4 arm (40.2%, risk ratio = 1.16; 95% CI: 0.99, 1.36) and the PoC CD4 + HH-IPT arm (39.9%, risk ratio = 1.15; 95% CI:0.99, 1.35), but with only weak evidence for an effect (p-values of 0.06 and 0.08, respectively). Unadjusted risk ratios and risk ratios adjusted for age and gender were very similar.

**Table 3:** HIV test uptake and yield of newly diagnosed HIV among TB contacts aged 14+ years.

	SoC arm (arm 1) n/N (%)	PoC CD4 arm (arm 2) n/N (%)	PoC CD4+ HH-IPT arm (arm 3) n/N (%)	Adjusted (for gender and age) arm 1 versus 2		Adjusted (for gender and age) arm 1 versus 3	
				PR (95% CI)	p-value	PR (95% CI)	p-value
HIV test uptake among:							
All contacts	304/876 (34.7%)	368/915 (40.2%)	357/895 (39.9%)	1.16 (0.99, 1.36)	0.060	1.15 (0.99, 1.34)	0.075
Newly diagnosed HIV-positive yield among:							
All contacts	26/984 (2.6%)	38/1032 (3.7%)	34/996 (3.4%)	1.40 (0.85, 2.30)	0.184	1.29 (0.78, 2.15)	0.320
Did not self-report HIV-positive	26/876 (3.0%)	38/915 (4.2%)	34/895 (3.8%)	1.41 (0.86, 2.32)	0.175	1.29 (0.77, 2.13)	0.332
Received HIV test	26/304 (8.6%)	38/368 (10.3%)	34/357 (9.5%)	1.20 (0.75, 1.94)	0.445	1.11 (0.69, 1.79)	0.672

SoC = standard of care; PoC = point-of-care; HH-IPT = household isoniazid preventive therapy; PR = prevalence ratio; CI = confidence interval.

### Yield of newly diagnosed HIV

The yield of newly diagnosed HIV among all contacts aged 14+years old was 2.6% in the SoC arm (Table 3). This was higher in the PoC CD4 arm (3.7% prevalence ratio = 1.40; 95% CI: 0.85, 2.30) and the PoC CD4 + HH-IPT arm (3.4%, prevalence ratio = 1.29; 95% CI: 0.78, 2.15), but with no statistical evidence for an effect (p-values of 0.18 and 0.32, respectively). Unadjusted and adjusted analyses showed very similar results. Results were also very similar if those who self-reported being HIV-positive were excluded from the denominators (Table 3). The yield of newly diagnosed HIV among those who received an HIV test was similar across the three arms (Table 3). The yield of all HIV (self-reported and newly diagnosed) among contacts aged 14+ years old was 13.6% in the SoC arm, 15.0% in the PoC CD4 arm and 13.6% in the PoC CD4 + HH-IPT arm.

### Linkage to care

Across all three arms, there were 98 newly diagnosed HIV positive contacts (from 93 households), in whom linkage into care and IPT initiation and continuation were evaluated. There were some imbalances in gender and age by arm, with the PoC CD4 + HH-IPT arm having the highest proportion male and lowest median age (Table 1).

**Fig 2:**
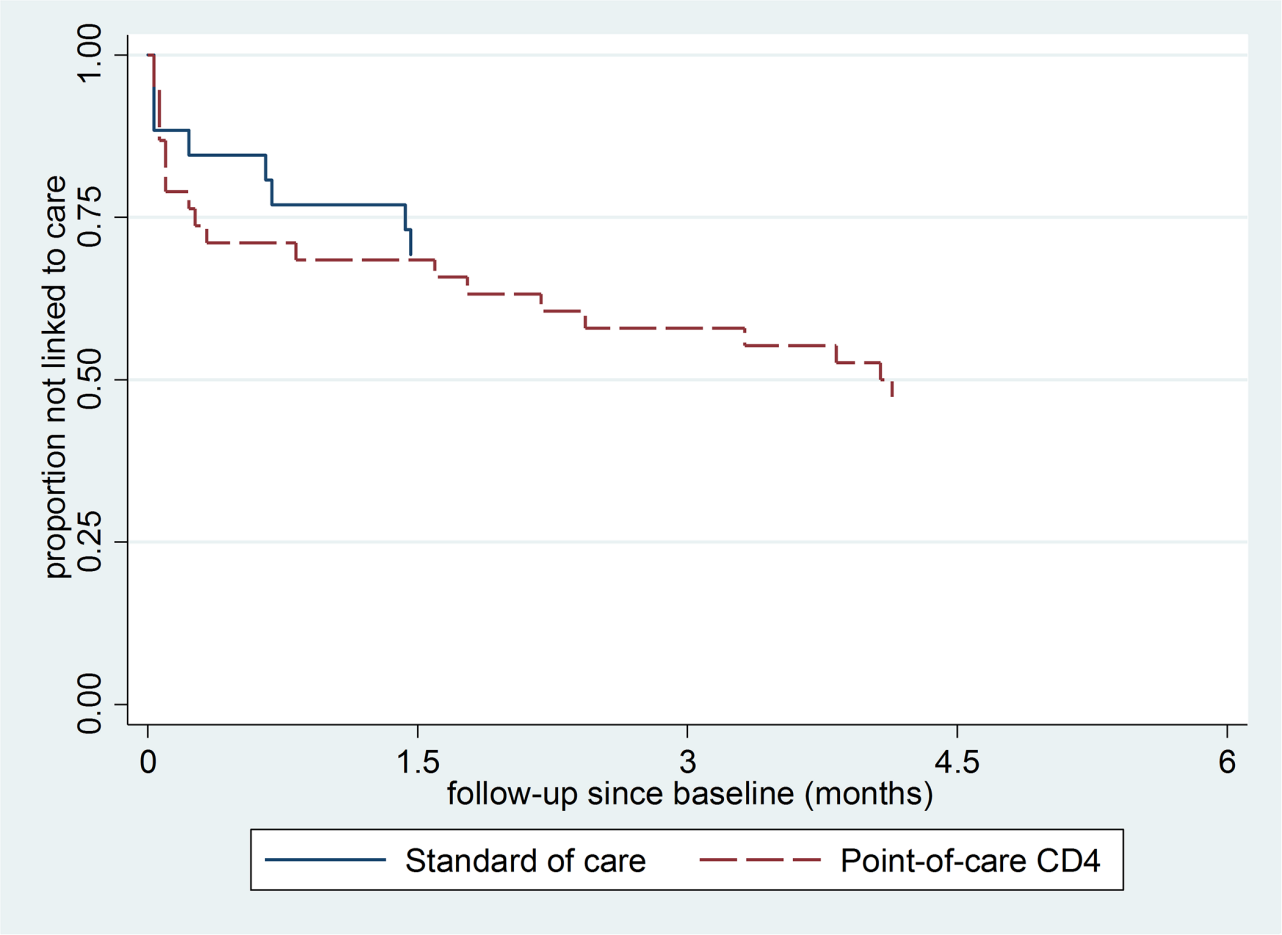
Kaplan-Meier plot of linkage-to-care by time since newly diagnosed HIV-positive, by study arm.

We recorded at least one study follow-up for 23 of 26 (88.5%) newly diagnosed HIV positive in the SoC arm, 34 of 38 (89.5%) in the PoC CD4 arm and 32 of 34 (94.1%) in the PoC CD4 + HH-IPT arm (Fig 1). Linkage to care within three months was recorded for 8 of 26 in the SoC arm (30.8%; Table 4). For the PoC CD4 arms, linkage was higher at 42.1% (risk ratio = 1.37; 95% CI: 0.68, 2.76), but with no statistical evidence of an effect (p-value = 0.38). Results were very similar when adjusted for gender and age, and when restricted to those for whom at least one study follow-up was recorded. Although the Kaplan-Meier plot (Fig 2) suggested that linkage to care may have been faster in the PoC CD4 arm compared to the SoC arm, there was again no statistical evidence for an effect (p-value from log-rank test = 0.30; Fig 2).

**Table 4:** Linkage to care within three months among TB contacts aged 14+ years who were newly diagnosed HIV-positive at baseline.

	SoC arm n/N (%)	PoC CD4 arm n/N (%)	Unadjusted		Adjusted (for gender and age)	
			RR (95% CI)	p-value	RR (95% CI)	p-value
Regardless of study follow-up	8/26 (30.8%)	16/38 (42.1%)	1.37 (0.68, 2.76)	0.382	1.48 (0.73, 2.99)	0.276
Restricted to those with at least one study follow-up	8/23 (34.8%)	16/34 (47.1%)	1.35 (0.68, 2.67)	0.385	1.43 (0.74, 2.79)	0.287

SoC = standard of care; PoC = point-of-care; RR = risk ratio; CI = confidence interval.

### Screening for tuberculosis

Among the 3,012 contacts aged 14+ years, 3,008 were screened for tuberculosis and 439 reported symptoms suggestive of tuberculosis (14.6%). We received laboratory results for 201 of these 439 (45.8%), of whom 15 (3.4%) had bacteriologically confirmed TB, giving an overall yield of TB of 0.5% (95% CI: 0.3%, 0.8%) in those screened. Comparing TB yield in the three arms showed nearly double the yield in the Standard of Care arm (0.71%) than in the PoC CD4 (0.39%) and PoC CD4 + HH-IPT arm (0.4%) but this was not statistically significant (p-value from Fisher’s exact test 0.557).

### Uptake and continuation of IPT

In the PoC CD4 arm, 20 contacts had a CD4 of at least 350, 18 of whom had at least one follow-up visit, at which three reported initiation of IPT. In the PoC CD4 + HH-IPT arm, 21 contacts had a CD4 of at least 350, 16 of whom were initiated on IPT in the household. The remaining five had at least one follow-up visit, at which four reported initiation of IPT. Hence, 20 of 21 contacts (95.2%) initiated IPT in the PoC CD4 + HH-IPT arm, compared to three of 20 contacts (15.0%) in the PoC CD4 arm (risk ratio = 8.20; 95% CI: 1.98, 33.95) with very strong statistical evidence of an effect (p = 0.004; p-value from Fisher’s exact test < 0.001).

Receipt of at least four months of IPT was reported for three of 20 contacts (15.0%) in the PoC CD4 arm, compared to 12 of 21 contacts (57.1%) in the PoC CD4 + HH-IPT arm (p-value from Fisher’s exact test = 0.009).

## Discussion

In our study, we aimed to evaluate the implementation of HIV testing, PoC CD4 testing and household IPT initiation in a HHCT program. We found poor uptake of HIV testing among contacts with an unknown or negative HIV status. The unexpectedly low uptake of HIV testing reduced the number of newly diagnosed HIV infected patients for analysis. Although there was some weak evidence that PoC CD4 testing led to a modest increase in HIV testing, it did not show an increased linkage to care among those who were not yet eligible for ART. Household IPT initiation was feasible and resulted in increased uptake and continuation.

Evidence from HHCT testing has reported a high prevalence of undiagnosed HIV [5,6] and had been suggested as a strategy to increase HIV testing uptake [17]. The HIV testing uptake in this study is comprehensively described elsewhere [18] but was lower than Shapiro *et al* who reported an uptake of 55% among household contacts. In contrast, other household-based HCT studies not provided as part of HHCT have achieved substantially higher HIV testing uptake; ranging from 69% to 99% [19]. We can postulate that improved uptake in dedicated household HCT activities might be a result of better outreach and community engagement which are not feasible or included in TB contact tracing. This highlights the challenge with “bundled” service delivery and potential trade-off in lower testing uptake compared to dedicated HCT delivery. However, an encouragingly high number of household members actually knew their status.

With the introduction of universal test and treat, regardless of CD4 count, POC CD4 becomes less relevant, however CD4 is still done to prioritise treatment and POC CD4 may still have played a role in encouraging people to present earlier. In our study we found that provision of Household PoCCD4 was feasible but did not significantly increase overall linkage to care. This study helps to resolve prior equivocal findings with more generalizable results indicating that POC CD4 at the time of HCT is not an effective way to markedly increase entry into care[13]. In addition, there was only weak evidence that an offer of PoC CD4, which was made at the beginning of the study visit in the two intervention arms, increased the probability that the household member would accept an HIV test.

Household IPT initiation for those who were not yet eligible for ART was feasible and resulted in increased IPT uptake and continuation. As the total numbers of eligible patients were low due to the high prevalence of known HIV-positivity and the lower uptake of HIV testing, it is difficult to comment on the value of such an intervention. Furthermore, the measurement of IPT uptake differed in the two arms, with it dependent on self-report after 10 weeks in the CD4 arm and study staff documentation in the CD4-IPT arm. We are confident that since self-report is more often associated with social desirability bias and patients were encouraged to attend for IPT, we do not believe the difference in reporting would have led to substantial under-reporting of IPT in the self-report arm. As we move towards a “‘test and treat”‘ strategy, the most appropriate point for IPT initiation will probably be in ART clinics once patients have stabilised on ART, rather than in the household.

The TB yield in this study was very low (0.5%) compared to previously published research from similar communities in South Africa which reported a prevalence of 1.5–5.9% amongst household contact[20–22]; there may be several explanations for this. As the primary outcomes of this pragmatic trial were dependent on HIV testing, the study teams were encouraged to focus on HIV testing. We believe TB screening and optimal sputum collection may thus have been compromised; which is supported by the higher TB yield in the SoC compared to the two intervention arms. This highlights the complexity associated with “bundled services”, which are intended to justify the high costs associated with a household visit, but ultimately affect the quality of interventions. We also only requested one spot specimen for Xpert testing on symptomatic patients rather than one spot and two early morning specimens for TB culture testing as described in previous studies [5,6]; the latter use of TB culture testing being particularly relevant given evidence which suggests reduced sensitivity of Xpert in certain settings[23] and more sensitive tests may be required for contact tracing populations. In addition, laboratory results were received for less than half of all presumptive TB contacts in our study, clearly reducing yield.

As one of the largest pragmatic randomised controlled trials of household TB contact tracing, the study was well randomised, adequate numbers were enrolled and follow up was recorded for 89/98 (91%) participants. The yield of newly diagnosed HIV was similar across all three arms; allowing for robust comparison across arms. However, higher numbers of household contacts already knew their HIV status and were on ART than originally anticipated. This, in addition to the high refusal rate of HIV testing in this household setting, resulted in low numbers being included in the evaluation of the two primary interventions; PoC CD4 and IPT initiation. South Africa has recently introduced universal test and treat for HIV but CD4 counts are still used to prioritise patients. The introduction of universal test and treat; may mean that IPT should be initiated in all patients on ART without contraindications and that health facilities would be more suitable for IPT initiation going forward.

## Conclusions

The results from this study suggest that the addition of HIV interventions in a TB household contact tracing programme should be done with caution and may compromise the efficiency of the TB case finding. We did not find evidence for promoting the use of household IPT or the use of POC CD4. Although feasible, these interventions had low impact due to the low uptake of HIV testing in households
